# Study of the Association between the Incidences of Congenital Anomalies and Hydrocephalus in Sudanese Fetuses

**DOI:** 10.5539/gjhs.v6n5p1

**Published:** 2014-04-27

**Authors:** Mustafa Z. Mahmoud, Hussien A. Dinar, Alsafi A. Abdulla, Esameldeen Babikir, Abdelmoneim Sulieman

**Affiliations:** 1 Radiology and Medical Imaging Department, College of Applied Medical Sciences, Salman bin Abdulaziz University, Al-Kharj, Saudi Arabia; 2 Basic Sciences Department, College of Medical Radiological Sciences, Sudan University of Science and Technology, Khartoum, Sudan; 3 National College for Medical and Technical Studies, Khartoum, Sudan; 4 Radiotherapy Department, College of Medical Radiological Sciences, Sudan University of Science and Technology, Khartoum, Sudan; 5 Radiological Sciences Department, College of Applied Medical Sciences, King Saud University, Riyadh, Saudi Arabia

**Keywords:** aqueduct stenosis, arnold-chiari malformation, dandy-walker malformation, hydrocephalus, ultrasound, spina bifida

## Abstract

This study was designed with an aim to detect the congenital anomalies appear to be linked to and in conjunction with hydrocephalus fetuses in Sudan, when ultrasound is used to exam fetuses in the second and third trimesters of pregnancy. This prospective cohort study was performed from December 2011 to December 2013, in a group consists of 5000 single gestation pregnant Sudanese women. In all cases, maternal ages were 35 years up to 48 years; mean age of 42.5 years. Pelvic; obstetric ultrasound scanning protocol used should meet the standards established by the American Institute of Ultrasound in Medicine (AIUM) for scanning in the second and third trimesters of pregnancy. Statistical Package for the Social Sciences (SPSS) was used to analyze the results. Diagnosed hydrocephalus cases (0.4%) were found to be associated with other fetal anomalies as aqueduct stenosis (45%), spina bifida (30%), Arnold-Chiari malformation (20%) and Dandy-Walker malformation (5%). The incidence of congenital anomalies and hydrocephalus in Sudanese fetuses showed considerable variation among different regions of Sudan. Hydrocephalus is associated with certain congenital anomalies. In agreement with previous studies, hydrocephalus is predominantly in male rather than female fetuses. The prevalence of fetal anomalies and hydrocephalus are within previously reported ranges.

## 1. Introduction

Hydrocephalus is defined as an increase in the cerebral ventricular size and/or sub-arachnoid space. It is caused by an imbalance between the production, circulation and resorption of cerebro-spinal fluid (CSF) (Tolmie, 2002). Hydrocephalus is characterized by a pathological accumulation of CSF in the ventricular system, causing pressure on the surrounding developing brain. If left untreated, the condition leads to various degrees of cognitive impairment, cerebral palsy and visual deficits. In severe cases the condition is fatal. In developed countries, most patients undergo surgical treatment, but the long-term prognosis is highly variable (Christensen, Hansen, & Garne, 2003; Persson, Hagberg, & Uvebrant, 2006; Lindquist, Persson, Fernell, & Uvebrant, 2010). Hydrocephalus is an important cause of neurologic morbidity and mortality in children. Although the widespread use of CSF shunting has reduced mortality, children with hydrocephalus often face multiple surgical procedures resulting in significant morbidity (Gupta et al., 2007; Wu, Green, Wrensch, Zhao, & Gupta, 2007).

The prevalence and demographics of hydrocephalus remain poorly defined, in part because the definition of “hydrocephalus” varies between studies; depending on the clinical criteria used to define hydrocephalus, the prevalence has been reported between 1 and 32 per 10 000 live births (Forrester & Merz, 2005; Dai et al., 2006; Persson, Anderson, Wiklund, & Uvebrant, 2007; Garne et al., 2010). Some studies have suggested that the prevalence of hydrocephalus increased between the 1960s and 1990s (Cavalcanti & Salomao, 2003; Fernell & Hagberg, 1998; Glinianaia & Rankin, 1999), possibly as a result of the increased survival of extremely preterm infants. Others have found that the prevalence of hydrocephalus diminished in recent years (Forrester & Merz, 2005; Persson et al., 2007; Fernell & Hagberg, 1998). The incidence of hydrocephalus accounts for approximately 50% of all forms of hydrocephalus (Schrander & Fryns, 1998).

The etiology of hydrocephalus is very heterogeneous. The majority consists of secondary forms, caused by intrauterine infections, intracranial hemorrhages, trauma, teratogens and tumors. Hydrocephalus may also result from neural tube defects and is found in association with other central nervous system malformations. Primary hydrocephalus occurs in 0.2 to 0.8 per 1000 live births (Chi, Fullerton, & Gupta, 2005). A possible genetic etiology is present in about 40% of patients with hydrocephalus. This includes cytogenetic abnormalities, monogenic or complex inherited conditions and multifactorial disorders. However, in the majority of cases the cause of primary hydrocephalus is still unknown (Verhagen et al., 2011).

As the resolution of the ultrasound equipment and the skill in ultrasound interpretation continue to improve, their role in prenatal diagnosis continues to expand. However, ultrasound findings in relatively large population studies have been demonstrated to have significant rates of false-positive and false-negative diagnosis. Accurate prenatal diagnosis, with an understanding of the limitations of diagnostic ultrasound, is required for appropriate counselling of parents, with regard to prognosis for the current pregnancy and risk of recurrence in the future. Realistic information regarding pregnancy outcome is necessary for both parents and medical staff to plan appropriate prenatal and neonatal management (Johns, Al-Salti, Cox, & Kilby, 2004).

This study was designed with an aim to detect the congenital anomalies appear to be linked to and in conjunction with hydrocephalus in Sudanese fetuses, when ultrasound is used to exam fetuses in utero, during the second and third trimesters of pregnancy.

## 2. Method

After the nature of the exam was fully explained in this prospective cohort study, the authors obtained written informed consent for all single gestations pregnant Sudanese women prior to each pelvic ultrasound scan. Our Institutional Review Board approved this study. In addition, a review and authorization of the study protocols were done by the Ethical Committee available at Sudan University of Science and Technology (SUST).

### 2.1 Selection and Description of Participants

From December 2011 to December 2013, one radiologist performed pelvic ultrasound scan and check for the presence of hydrocephalus and its related fetal anomalies in a group of 5000 pregnant Sudanese women. In all cases, maternal ages were 35 years up to 48 years; mean age of 42.5 years, and presented with a single gestation, during the second and third trimesters of pregnancy.

Participant recruitment, according to their Sudanese nationality, area of location Sudan, presence of fetuses hydrocephalus conditions confirmed by ultrasound examination, ages and ethnicities. Because of Sudan’s ethnic diversity remained one of the most complexes in the world, participants were divided into five ethnic groups in which (n = 2350; 47%) from Center of Sudan, (n = 1600; 32%) from North of Sudan, (n = 600; 12%) from West of Sudan, (n = 250; 5%) from East of Sudan, and (n = 200; 4%) from South of Sudan. Participants were scanned in the Ultrasound Department, College of Medical Radiological Sciences (CMRS) - SUST.

### 2.2 Ultrasound Equipmet

Pelvic ultrasound was performed using a high-resolution general electric (GE) ultrasound medical system, logic 5 expert ultrasound unit equipped with a 3.75 MHz convex probe, model 2302650 with serial number of 1028924YM7, manufactured date in 2005 and made by the Yokogawa medical system, Ltd. 7-127 Asahigaoka 4-Chome Hino-Shi, Tokyo, Japan. Printing facilities were issued through the ultrasound, digital graphic printer, 100 V; 1.5 A; and 50/60 HZ, with the serial number of 3-619-GB1-01 and made by Sony Corporation- Japan.

### 2.3 Ultrasound Technique

Fetal ultrasound was performed only when there is a valid medical reason, and the lowest possible ultrasonic exposure settings used to gain the necessary diagnostic information (Barnett et al., 2000; Bly & Van den Hof, 2005).

Fetal anatomy was adequately assessed by ultrasound after approximately 18 weeks’ gestational (menstrual) age. It may be possible to document normal structures before this time, although some structures can be difficult to visualize due to fetal size, position, movement, abdominal scars, and increased maternal abdominal wall thickness (Phatak & Ramsay, 2010). A second or third trimester scan may pose technical limitations for an anatomic evaluation due to imaging artifacts from acoustic shadowing.

The following areas: head, face, and neck: lateral cerebral ventricles; choroid plexus; mid-line falx; cavum septi pellucidi; cerebellum; cistern magna; and upper lip, of assessment represent the minimal elements of a standard examination of fetal anatomy. A more detailed fetal anatomic examination was considered necessary if an abnormality or suspected abnormality is found on the standard examination (American Institute of Ultrasound in Medicine, 2013).

Adequate documentation is essential for high quality patient care. A permanent record of the ultrasound examination and its interpretation and images of all appropriate areas, both normal and abnormal, were recorded. Variations from normal size were accompanied by measurements. Images were labeled with the patient identification, facility identification, examination date, and side (right or left) of the anatomic site imaged. An official interpretation (final report) of the ultrasound findings was included in the patient’s medical record. Retention of the ultrasound examination was consistent both with clinical needs and with relevant legal and local health care facility requirements (Chervenak & Chervenak, 2007).

Diagnostic ultrasound studies of the fetus are generally considered safe during pregnancy (Doubilet & Benson, 2010). This diagnostic procedure was performed only when there is a valid medical indication, and the lowest possible ultrasonic exposure setting was used to gain the necessary diagnostic information under the ALARA (as low as reasonably achievable) principle (Marinac-Dabic, Krulewitch & Moore, 2002; Miller, Brayman, & Abramowicz, 1998; Sheiner, Freeman, & Abramowicz, 2005). A thermal index for soft tissue (TIS), and a thermal index for bone (TIB) were used when bone ossification is evident. In keeping with the ALARA principle, motion mode (M-mode) imaging was used instead of spectral Doppler imaging to document fetal heart rate (US Food and Drug Administration, 2012).

### 2.4 Statistical Analysis

For the statistical analysis, Microsoft Excel Software and Statistical Package for the Social Sciences (SPSS) (SPSS Inc., Chicago, IL, USA) version 15 for windows were used. The demographic distribution of hydrocephalus occurrence in Sudanese fetuses and its related congenital anomalies were shown in a form of comparison tables. The difference between ethnic groups was checked by one-way analysis of variance (ANOVE). *P* value terms such as equal and less to be used for significance; *P* value (*P* ≤ 0.001) was considered to be significant.

## 3. Results

**Figure 1:**
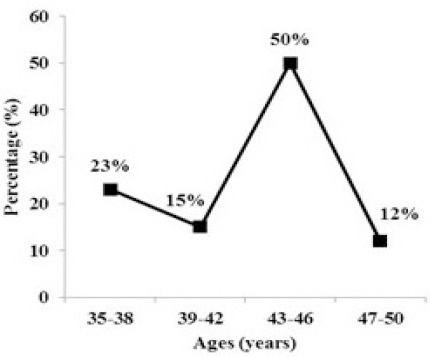
The age distribution of the study samples

Twenty fetuses with hydrocephalus were diagnosed during the study period, giving an overall prevalence of 0.4% per 5000 cases of intrauterine pregnancy in Sudanese fetuses. Of the Scanned participants, a total of 2500 subjects were in the age group 43-46 years, representing (50%) of the population. The age group of 47-50 years was the smallest (12%) of the population (Table 1). The highest mean ± SD of ages was (48.5 ± 2.5) years found in the age group 47-50 years while the lowest mean ± SD of ages was (36.5 ± 2.5) years found in the age group 35-38 years (Table 1 and Figure 1).

**Table 1:** The age distribution of the study samples

Ages (years)	Frequency	Percentage	Mean age ± SD (years)
35-38	1150	23%	36.5 ± 2.5
39-42	750	15%	40.5 ± 2.5
43-46	2500	50%	44.5 ± 2.5
47-50	600	12%	48.5 ± 2.5
*P* value	-	-	0.001
Total	5000	100%	42.5 ± 0.0

**Figure 2:**
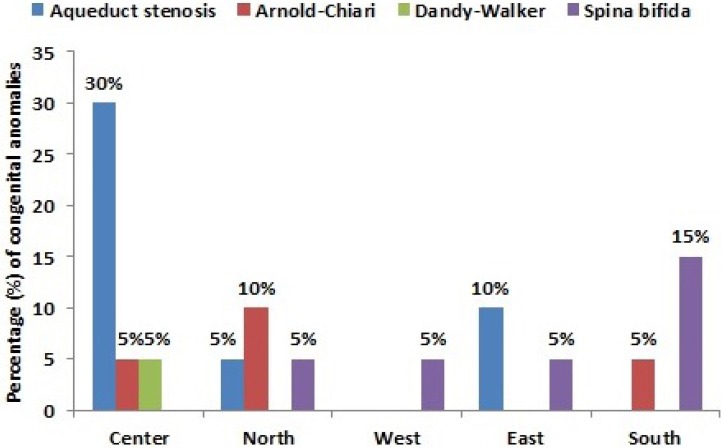
The percentage of common congenital anomalies associated with hydrocephalus in different Sudanese ethnics

The incidence of hydrocephalus (n = 20; 0.4%) was higher in male fetuses (n = 11; 55%) in comparing to female fetuses (n = 9; 45%); *P* ≤ 0.001. The diagnosed cases of hydrocephalus were found to be associated and conjoined with other pathological anomalies such as: Aqueduct stenosis (n = 9; 45%), spina bifida (n = 6; 30%), Arnold-Chiari malformation (n = 4; 20%) and Dandy-Walker malformation (n = 1; 5%) (Table 2 and Figure 2).

**Table 2:** Incidence of hydrocephalus and its related anomalies in Sudanese fetuses

Ethnics	Ethnics (Frequency and percentage)	Fetuses with hydrocephalus (Frequency and percentage)	Anomalies in relation with hydrocephalus (Frequency and percentage)
Center of Sudan	(n=2350; 47%)	(n=12; 60%)	Aqueduct stenosis (n=6; 30%)
			Arnold-Chiari (n=1; 5%)
			Dandy-Walker (n=1; 5%)
North of Sudan	(n=1600; 32%)	(n=3; 15%)	Aqueduct stenosis (n=1; 5%)
			Spina bifida (n=1; 5%)
			Arnold-Chiari (n=2; 10%)
West of Sudan	(n=600; 12%)	(n=2; 10%)	Spina bifida (n=1; 5%)
East of Sudan	(n=250; 5%)	(n=2; 10%)	Aqueduct stenosis (n=2; 10%)
			Spina bifida (n=1; 5%)
South of Sudan	(n=200; 4%)	(n=1; 5%)	Spina bifida (n=3; 15%)
			Arnold-Chiari (n=1; 5%)
Total	(n=5000; 100%)	(n=20; 100%)	(n=20; 100%)

For ethnic consideration, females from Center of Sudan show the highest incidence of hydrocephalus (n = 12; 60%) and its related anomalies in the scanned fetuses while females from the South of Sudan show the lowest incidence of hydrocephalus (n = 1; 5%) and its related anomalies (Table 2 and Figure 2).

## 4. Discussion

Previous investigations on familial aggregation of hydrocephalus are very few. A recurrence risk of hydrocephalus of 1.4% among younger siblings of 205 cases was reported (Burton, 1979). More recently, Varadi, Toth, Torok and Papp (1988) found a sibling recurrence risk of 4% in the younger siblings of 261 affected cases. A recurrence risk for siblings as the only type of relative is rather inconclusive regarding whether the observed increased recurrence risk may have been due to genetic factors or shared maternal related risk factors. Our findings were based on 5000 cases and authors were not able to compare the risk of hydrocephalus for individuals with an affected relative to the risk for individuals with known, unaffected relatives of the same type. This study found a prevalence of hydrocephalus at 0.4 per 5000 scanned fetuses. Prevalence of hydrocephalus has previously been reported to be 3.7 per 10000 births in a Northern Region in the United Kingdom 1985-1996 (Glinianaia & Rankin, 1999), and 8.1 per 10000 births in Strasbourg, France 1979-1987 (Stoll, Alembik, Dott, & Roth, 1992). In the French study a large proportion of cases (29%) were diagnosed in infancy (after 1 week). As the definition of hydrocephalus is broad without strict criteria for the size of the ventricles, prevalence may differ between studies. The British study (Glinianaia & Rankin, 1999) showed a significant increase in total prevalence over time due to increased prenatal detection while the live birth prevalence remained stable. Fetal deaths that previously remained undiagnosed may have been diagnosed prenatally and terminated. Live birth prevalence in the French study was 4.8 per 10 000 births, in the British study (Stoll et al., 1992), 2.5 per 10 000 births compared to 0.4 per 5000 fetuses in our study.

The difference between ethnic groups was checked by one-way analysis of variance (anova) and was considered statistically significant (*P* = 0.001), also his study found a high proportion of associated malformations, such as: Aqueduct stenosis (n = 9; 45%), spina bifida (n = 6; 30%), Arnold-Chiari malformation (n = 4; 20%) and Dandy-Walker malformation (n = 1; 5%) in the diagnosed hydrocephalus cases (n = 20; 100%). This high proportion of associated anomalies was comparable to what was found for omphalocele, where about half of the cases have associated anomalies (Calzolari, Bianchi, Dolk, & Milan, 1995). Other studies of hydrocephalus have also shown this high proportion of associated malformations and karyotype anomalies (Glinianaia & Rankin, 1999; Stoll et al., 1992). This emphasizes the importance of looking for other malformations after a prenatal or postnatal diagnosis of hydrocephalus. Studies of major malformations of other organ systems have shown the impact of associated malformations and karyotype anomalies on birth outcome and survival (Garne, Stoll, & Clementi, 2001; Garne et al., 2002; Garne, Loane, Wellesley, & Barisic, 2009).

The recent decline in the overall rate of neural tube defects, including spina bifida in the United States (US) has been attributed to increased folic acid supplementation (Williams et al., 2002). The US Public Health Service first recommended folic acid supplementation in 1992 and the Institute of Medicine followed in 1998. A meta-analysis of studies from 1965 to 2005 found a modest decrease in risk of overall hydrocephalus with multi vitamin and folate supplementation (Goh, Bollano, Einarson, & Koren, 2006). In this Study, results confirmed a decrease in the prevalence of spina bifida in Center of Sudan and there are higher incidences in Southern Sudan. Of note, even in countries where folic supplementation has not been implemented, improved prenatal diagnosis leading to pregnancy termination is thought to have decreased the prevalence of hydrocephalus related to spina bifida (Sípek, Gregor, Horácek, & Masátová, 2002; Persson et al., 2007).

Obtained results confirm that hydrocephalus is a male predominance (n= 11; 55%) rather than female fetuses (n= 9; 45%); *P* ≤ 0.001, which has been noted in other studies of hydrocephalus (Glinianaia & Rankin, 1999; Forrester & Merz, 2005; Persson et al., 2007) but only achieved statistical significance in one (Fernell, Hagberg, Hagberg, & von Wendt, 1986).

A potential limitation of this study was incomplete registration of the specific sub types of hydrocephalus. Thus, it is possible that some of the findings could result from ascertainment bias. Despite this limitation, our large sample size and population based data allowed us to determine demographic risk factors for hydrocephalus. Also, further follow-up of the development and needs of children with hydrocephalus surviving infancy is needed.

In conclusion, the incidence of hydrocephalus in Sudanese fetuses was 0.4% per 5000 scanned fetuses. Ethnically females from Center of Sudan show the highest incidence of hydrocephalus (n = 12; 60%) and its related anomalies in the scanned fetuses while females from South of Sudan show the lowest incidence of hydrocephalus (n = 1; 5%) and its related anomalies. Increases the incidence of Hydrocephalus and its related anomalies in center of Sudan rather than other regions, seems to be due to the proportion of the great exodus of the Sudanese population in the recent years towards the Center especially Khartoum, Sudan’s capital as a result of the availability of health services and other services rather than the periphery.
